# Eye Tracking in the Wild: Piloting a Real-Life Assessment Paradigm for Older Adults

**DOI:** 10.16910/jemr.12.1.4

**Published:** 2019-05-24

**Authors:** Damaris Aschwanden, Nicolas Langer, Mathias Allemand

**Affiliations:** Florida State University, USA; University of Zurich, Switzerland

**Keywords:** feasibility study, real life, eye movements, eye tracking, personality, individual differences, grocery shopping, usability, older age

## Abstract

Previous research showed associations between personality traits and eye movements of young adults in the laboratory. However, less is known about these associations in real life and in older age. Primarily, there seems to be no paradigm to assess eye movements of older adults in real life. The present feasibility study thus aimed to test grocery shopping as a real-life assessment paradigm with older adults. Additionally, possible links between personality traits and eye movements were explored. The sample consisted of 38 older individuals (M = 72.85 years). Participants did their grocery shopping in a supermarket while wearing an eye tracker. Three key feasibility issues were examined, that is (1) wearability of the eye tracker during grocery shopping, (2) recording, and (3) evaluation of eye movements in a real-life context. Our real-life assessment paradigm showed to be feasible to implement and acceptable to older adults. This feasibility study provides specific practical recommendations which may be useful for future studies that plan to innovatively expand the traditional methods repertoire of personality science and aging research by using eye tracking in real life.

## Introduction

Moving the eyes is one of the key ways through which humans gather information about the world around them. Consequently, eye tracking has become an important method to investigate eye movements in different areas of psychology such as cognitive, neuropsychological, developmental and personality science [1, 2, 3]. Across all these research areas, it has been of interest to identify factors that shed light on different eye movement patterns. Generally, visual exploration is driven by two main factors, that is the stimuli of the environment as well as personal interests and intentions [4, 5]. One factor that belongs to the latter category and has received relatively little attention is personality [6, 7]. Specifically, what is the role of individual differences in personality for eye movements of older adults? For example, do older individuals who are more open to experience look at more different things when they inspect their surroundings? Do older individuals who are high on extraversion look more often at other people? What follows is a brief overview of previous research concerning personality and eye movements to introduce the theoretical background in which our real-life assessment paradigm is relevant.

### Laboratory-Based Eye Tracking Studies

Previous research showed significant associations be- tween different personality traits and eye movements of young adults in the laboratory. For instance, it has been suggested that individual differences in personality such as anxiety [8] or loneliness [9] influence various forms of attention (e.g., task switching or gaze- triggered orienting; see also Kaspar and König [6], for a review). With regard to the Big Five personality traits, participants with higher levels of openness showed increased durations of fixations to the eyes of an individual who sat opposite the participants [10]. According to the authors, a possible explanation may be that individuals who show higher levels of openness attempt to obtain information from the other person. In a car advertisement study, neuroticism was positively related to the duration and number of fixations on cars, but negatively related to the duration and number of fixations on price and text [11]. Rauthmann, Seubert, Sachse, and Furtner [12] suggested that individuals with high levels of neuroticism might take longer in processing complex stimuli because they try to validate their value to prevent themselves from potential harm (e.g., doubtful cars). Furthermore, curiosity has been revealed as a robust and reliable predictor of an individual’s eye movement behavior in laboratory scene-viewing of buildings, interiors, and landscapes [7]. This means, participants with higher levels of curiosity showed higher levels of exploratory behaviors (i.e., higher number of regions visited) in the scene-viewing task. In general, the effect sizes of the results reported in literature range from small to medium [12].

### Eye Tracking in the Wild

A search of the literature revealed few studies that investigated the associations between personality traits and eye movements in the wild or in real life, respectively. Hoppe, Loetscher, Morey, and Bulling [13] examined whether curiosity could be predicted based on natural eye movements during a real-world task. It should be noted that curiosity was examined as the outcome here, whereas curiosity was used as the predictor in the study of Risko and colleagues [7]. Hoppe et al.’s participants ( *N* = 26) were given AU$5 to go to one of the shops on campus and buy an item of their choice, which they were allowed to keep or eat while wearing a mobile eye tracker. After 10 to 15 minutes, the participants returned to the laboratory and filled in two curiosity questionnaires. For 11 of 26 participants, Hoppe and colleagues [13] predicted the correct class of curiosity out of up to four classes (depending on the curiosity scale as they used more than one scale to assess curiosity). In 2018, the same research group expanded their work by tracking the eye movements of 42 participants and including the prediction of the Big Five personality traits [14]. This time, they predicted four of the Big Five personality traits (neuroticism, extraversion, agreeableness, conscientiousness) and perceptual curiosity from natural eye movements. Their findings demonstrate a considerable influence of personality on everyday eye movement control. Apart from Hoppe et al. [13, 14], there is a general lack of research of the associations between personality traits and natural eye movements in real life.

While the aforementioned studies relied on (under- graduate) student samples, future work should expand previous knowledge by exploring these associations in older adults. Investigating these links in daily life of older adults is important, because it helps to better understand how older people are and how they gaze, and how these associations are manifested in daily life and not only in the laboratory [15, 16]. Furthermore, using eye tracking in personality research expands the traditional methods repertoire of self-reports and behavioral observations [17]. It seems particularly worthwhile to use eye tracking as an objective method in aging research, because older age is a phase that is particularly susceptible to individual and environmental changes and non-normative events [18]. If multiple measurement occasions are sampled, it may be that older individuals tend to change their internal standards of perceptions due to the accompanying changes that aging brings with it, and this may impair the interpretation of study results [19]. Although research may establish measurement invariance to consider this issue, objective methods such as eye tracking may be an interesting and innovative alternative. Moreover, knowing the associations between personality traits and natural eye movements in real life may not only be interesting for aging research, but also for marketing research as a general demographic shift to older populations is indispensable due to the increase of global life expectancy [20].

### Innovation: Testing a Real-Life Paradigm

Before researchers can start to investigate the associations between personality traits and eye movements in real life, it is required to test and establish an appropriate real- life assessment paradigm. Inspired by the work of Hoppe and colleagues [13, 14], we undertook a feasibility study to pilot grocery shopping as a real-life assessment paradigm with older adults. Grocery shopping was chosen because of two reasons. On the one hand, we aimed to ex- pand Hoppe et al.’s work by implementing a real-life assessment paradigm (i.e., grocery shopping) rather than a real-life task (i.e., shopping task). This means, our participants were allowed to do their individual grocery shopping amounting to 30 Swiss Francs (approximately $30), whereas Hoppe et al.’s participants were given AU$5 to purchase a drink or confectionary. Establishing such a real-life assessment paradigm is important for the following reasons: (a) its ecological validity, (b) because it includes an objective measurement method, and (c) the task is familiar to older participants. On the other hand, shopping can be considered as one of the most important activities to maintain elderly people’s independent daily life [21, 22].

As this feasibility study introduces a simple, but innovative real-life assessment paradigm with older adults, it must address three key feasibility issues. First, will older adults wear the eye tracker all the time during grocery shopping? Our real-life assessment paradigm required that participants do not remove the eye tracker in order to rec- ord data. Second, is it possible to successfully record eye movements of older adults in a grocery store? There are several factors that might influence the recording of eye movements in an unstandardized setting, for example the illumination, participants’ free movements, and the vary- ing distance to the shelves. Third, is it possible to evaluate the eye movements with respect to three areas of interest? Unlike laboratory-based eye tracking studies, this feasibil- ity study did not use markers that facilitate the eye tracker data analysis. In contrast, all videos of the scene camera had to be coded manually by two independent coders (see Lappi [23] for an overview of special concerns that naturalistic research brings about).

In addition, we explored whether there are associations between different personality traits and eye movements (i.e., number of fixations on three areas of interest) during grocery shopping. However, it is important to note that these exploratory analyses provide preliminary results and are available in the supplementary file only, because the sample size of this feasibility study limits the power to de- tect links between personality traits and eye movements. In particular, our focus was on piloting the real-life assess- ment paradigm as this is one of the first attempts to assess eye movements of older adults in real life.

## Methods

### Participants

For the present feasibility study, a total of 38 healthy older individuals (79% female) were recruited via an ad- vertisement in a magazine for older adults as well as a da- tabase of older adults who are interested in study partici- pations. Participants met the following inclusion criteria: fluent German or Swiss German speaker, full mobility, normal or corrected-to-normal vision, and no psychiatric or neurologic diseases. The mean age of the sample was 72.85 years (*SD* = 7.25, range = 59-87 years). The mean level of education was 5.89 on a scale from 1 = no educa- tion to 7 = university. All participants had a Mini Mental State Examination (MMSE [24]) score higher than 26, and thus did not show signs of cognitive impairment (MMSE scores < 24). All methods and procedures were approved by the ethics committee for psychological and related research of the University of Zurich. The participants gave their written informed consent prior to study participation.

### Procedure

Participants came to the laboratory and completed several questionnaires (e.g., sociodemographic, health, personality) and cognitive tasks (e.g., MMSE). Next, they walked to a local supermarket (Coop Center Eleven Oer- likon) accompanied by a student assistant. In the super- market, the eye tracker was calibrated, and participants were given 30 Swiss Francs (approximately $30) to do their grocery shopping. Participants were allowed to buy food and non-alcoholic drinks. While participants did their grocery shopping, a student assistant waited in the entrance hall of the supermarket, monitoring the laptop to which the eye tracker data was sent in real-time. After the shopping, they had to answer six questions regarding their behavior during grocery shopping to check for possible reactivity effects (i.e., censored or artificial behavior). They were also asked whether they have ever been in this supermarket before their study participation. Furthermore, the receipts were collected by the student assistant to know what participants bought. Participants were allowed to keep the items they bought. On average, participants shopped for 10.55 minutes (*SD* = 4.34, range = 4-21 minutes). Correlations between shopping duration and personality traits ranged from *rs* = -.34 (95% CI [-.683, .093], *p* = .087) to *rs* = .23 (95% CI [-.171, .570], *p* = .250).

### Legal and Ethical Considerations

Real-life assessment paradigms that capture infor- mation about bystanders must deal with legal and ethical concerns [25, 26]. In the present study, bystanders were other shoppers who were recorded by the scene camera of the eye tracker if the participants looked at them. We considered several procedures to legally and ethically implement our feasibility study. First, the permission to conduct the study was given by the supermarket management. Considering legal issues, the video recordings of the scene camera are unfixed and thus do not violate the right to one's own image (i.e., an individual’s right to control the use of his or her image, including the right to refuse publication thereof) in Switzerland. Considering ethical issues, we implemented the following safeguards to protect potential bystanders’ privacy and ensure the confidentiality of the data. First, video recordings of bystanders will not be published at any time to comply with the right to one's own image. Second, the coders do not code the videos if they know bystanders in the video. Third, if a participant asks for his or her video recording after study participation, all bystanders are cut out of the video. It is thus highly unlikely that the real-life assessment paradigm as we have established it violates privacy rights of people who are inadvertently recorded.

### Measures

**Figure 1.**
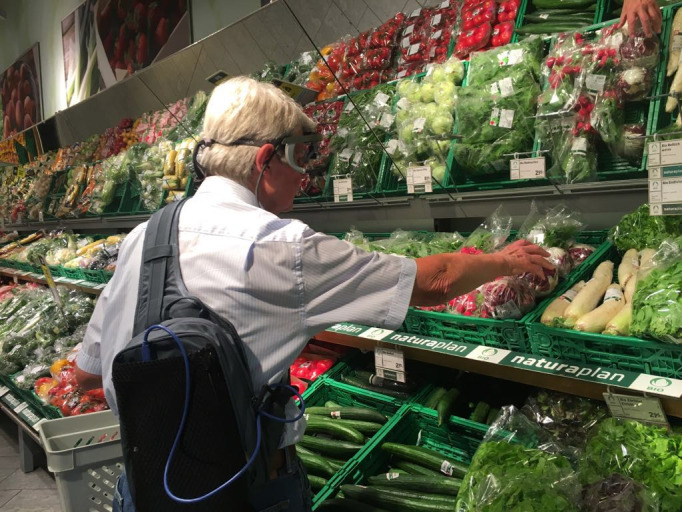
The eye tracker glasses were connected to a tablet that was stored in a small backpack carried by the participant. This participant kindly agreed to include this picture in scientific presentations and publications.

The wireless Dikablis Professional Eye Tracking Glasses and the corresponding software D-Lab Version 3.0 from Ergoneers [27] were used to assess eye movements. Binocular gaze data was recorded at 60 Hz, and the scene video camera recorded on full high definition (HD) resolution (1920 x 1080 pixel). The accuracy for pupil detection provided by Ergoneers is 0,05°, and 0,1°-0,3° for glance direction. However, this may not correspond to the actual accuracy during grocery shopping and is thus discussed as a limitation (see “Discussion”). A wide-angle lens was put on the scene camera, which allowed more of the scene to be included in the video. The eye tracker glasses were connected to a tablet that was stored in a small backpack carried by the participants (see Figure 1). Data was stored on this tablet and sent via WLAN to a laptop in real-time. WLAN was provided by a router. The eye tracker was calibrated before participants did their grocery shopping. We analyzed the number of fixations on different areas of interest, because a meta-analysis has shown that the number of fixations is one of the most widely used parameter [28]. Fixations are defined as pauses over informative areas of interest [29]. Using D-Lab 3.0 [27], the fixations were calculated according to the principle of Salvucci and Goldberg [29].

Because it is critical to estimate how obtrusive the real-life assessment paradigm is, we checked for possible reactivity effects of the participants. Directly after the shopping, participants had to indicate whether or not they behaved (a) openly, (b) curiously, (c) interestedly, (d) stressfully, or (e) nervously during the grocery shopping. Optionally, they could report further behaviors in an open answer format.

The Big Five personality traits (openness, neuroticism, conscientiousness, extraversion, and agreeableness) were measured using the Big Five Inventory [30]. The 45 items were rated on a 7-point Likert scale ranging from 0 (strongly disagree) to 6 (strongly agree). Moreover, the personality trait curiosity was assessed using the Curiosity and Exploration Inventory (CEI-II [31]). This survey consists of two subscales, that is exploration (four items) and absorption (three items). Exploration reflects an orientation toward seeking novel and challenging objects, events, and ideas. Absorption reflects the ability to self-regulate attention to allow for immersion in activities. The total of seven items were rated on a 4-point Likert scale ranging from 1 (strongly disagree) to 4 (strongly agree). The internal consistencies (Cronbach’s alpha) of all personality measures ranged from acceptable (α = .71; curiosity) to good (α =.88; extraversion).

### Coding of the Video Material

The centerpiece of the present feasibility study was on coding the video material. The videos from the scene camera could not be coded automatically because the eye tracker data were collected in the wild (i.e., real-life con- text), having no standardized conditions and thus disclaiming to use markers that usually facilitate the eye tracker data analysis. Hence, the most time-consuming part of the feasibility study was to code the scene videos. All videos were watched and coded in slow motion by two independent coders. The coders completed several training sessions before coding the video material of this feasibility study. The videos were coded using the eye tracker software D- Lab 3.0 provided by Ergoneers.

Derived from previous research, we defined two areas of interest; (a) different products [7, 13, 14] and (b) price tags [11]. In addition, we defined the products that were actually bought as a third area of interest. Different products were defined as “all products in the supermarket”. Price tags were defined as “price tags on the product itself or on the shelves”. The products that were actually bought were defined as “products that were in the shopping basket and paid based on the receipts”. This means, participants’ receipts were checked and compared with the video material to clearly identify the products that were in fact bought. This was necessary because some participants did not fixate on the product while putting it in their shopping basket. Hence, the scene camera did not record this fixation and it is theoretically possible that participants put the product back in the shelf instead in the shopping basket. To exclude this theoretical possibility, receipts were checked.

Next, heatmaps were used to visualize fixations lasting 100ms or longer [12]. The heatmaps were shown time based (100ms), so they did not overlay for fixation visualization. The spot radius (size of heatmap) was set to 35 pixels. Subsequently, two independent coders counted the number of fixations (shown as red dots) for each area of interest separately.

After the videos were coded, the number of fixations on different area of interest were corrected for the individual shopping duration and the individual total number of fixations during grocery shopping. As described before, participants differed in their shopping duration. Furthermore, it seems possible that some participants looked at one of the three areas of interest all the time during grocery shopping, whereas others might have looked at the areas of interest less frequently in relation to their total number of fixations during grocery shopping. Hence, we calculated the mean of number of fixations on the areas of interest per minute (MNFminAOI), divided it by the total number of fixations per minute (NFmin), and used this measure for the analyses (NFcorr; Equation 1). To calculate the mean of number of fixations on the areas of interest per minute (MNFminAOI) (Equation 2), the number of fixations on the areas of interest (NFAOI) was divided by the total number of fixations per minute (NFmin). To compute the total number of fixations per minute (NFmin), the total number of fixations (NFtot) was divided by the shopping duration in minutes (Dmin) as displayed in Equation 3. The data set is available upon request.

NFcorr = MNFminAOI / NFmin (1)

MNFminAOI = NFAOI / NFmin (2)

NFmin = NFtot / Dmin (3)

Statistical Analyses

Power calculations were conducted using the ‘‘pwr” package [32] in R [33]. Of the 38 participants, *n* = 10 (26.3%) were excluded from exploratory data analyses (i.e., correlations) due to missing eye tracker data (note that the reasons for missing eye tracker data are further described in the section “feasibility” of the results). Referring to prior work, the correlation coefficients of the associations between personality traits and eye movements range from *r* = .05 - .30 in laboratory-based studies [12]. The power analysis revealed that a sample size of 84 to 3,136 participants would be needed to achieve enough power to detect correlation coefficients between *r* = .05 - .30. The present sample provides power of 6% to estimate a correlation coefficient of .05 at the 5% significance level, and a sample size of 3,136 participants would be needed to achieve 80% power for this value. If true correlation coefficients are .30, the power estimate would be 35%. A sample size of 84 participants would be needed to achieve 80% power for this value. Based on the given sample size, it is possible to detect effects that are >.50 ( *n* = 28, significance level = 0.05, power = 80%). The present feasibility study is underpowered to investigate the rather weak associations between personality traits and eye movements, but we conducted them for exploratory purposes. Spearman correlations (nonparametric data) were performed to examine the associations between personality traits and the number of fixations. These exploratory data analyses were based on *n* = 28 (75% female). The analyses did not include covariates because the present study piloted the feasibility of grocery shopping as a real-life assessment paradigm. No significant correlations between personality traits and eye movements were found. These results are available in the supplementary file.

## Results

### Feasibility

To determine if it realistic to use our real-life assessment paradigm with older adults, we examined three key feasibility issues. First, 37 participants wore the eye tracker all the time during grocery shopping. One participant decided against wearing the eye tracker during grocery shopping. This participant wore the eye tracker during the calibration process, but then felt too odd to wear it for the real-life assessment paradigm. Thus, 37 out of 38 participants completed the real-life assessment paradigm (attrition rate: 2.6%). Furthermore, we checked for possible reactivity effects of the participants. We addressed this issue by analyzing self-reported obtrusiveness (see “Measures”). Of the participants, 42.9% behaved openly and curiously, 46.4% behaved interestedly, 25.0% behaved stressfully, and 21.4% behaved nervously. The participants did not report any outstanding behaviors in the open answer format. Although there might be some minor reactivity effects for some participants, the real-life assessment paradigm seems to be unobtrusive and does not interfere much with the normal shopping behavior of the participants.

Second, it was possible to successfully record eye movements in a grocery store for 28 of 37 participants (75.7%). The reasons for missing eye tracker data were technical problems such as the scene camera was not recording (55.6%), the scene video was frozen (33.3%), or the connection cable from the eye tracker glasses to the tablet was displayed (11.1%).

Third, is was possible to evaluate the eye movements with respect to three areas of interest for 28 of 28 available videos (100%). The video coding was very time-intensive because no markers were used. Specifically, it took approximately 24 hours to code a video of 10 minutes for one coder. Estimates of intercoder agreement were obtained by calculating Krippendorff’s Alpha [34]. The intercoder reliability was acceptable (>.60) for all areas of interest (number of fixations on different products = .77; number of fixations on price tags = .61; and number of fixations on products bought = .71).

### Proportions

In addition, we analyzed descriptively the proportions of the number of fixations on the three different areas of interest (proportion 1 = different products / price tags; proportion 2 = different products / bought products; proportion 3 = price tags / bought products) to descriptively compare them. On average, participants looked 7.58 times more at different products in relation to price tags (*SD*: 7.98). They also looked 9.53 times more at different products in relation to the products they actually bought (*SD*: 7.65). However, the proportion between price tags and bought products was rather small, participants looked 1.67 times more at price tags than at bought products ( *SD*: 1.85).

## Discussion

The present feasibility study aimed to assess eye movements using a real-life assessment paradigm. We successfully piloted grocery shopping as a real-life assessment paradigm with older adults. The current assessment paradigm was feasible for 97.4% of participants. Furthermore, it was possible to record eye movements for 75.7% of participants, and 100% of the videos could be coded. Thus, there is preliminary support for the use of grocery shopping as a real-life assessment paradigm with older adults. However, an issue that was not addressed in this study was actual accuracy of pupil detection during grocery shopping. We thus cannot exclude that the scene camera moved during grocery shopping, causing errors in gaze position in scene image coordinates. This means, gaze data with poor accuracy can focus on close-by areas of interests instead of the one gazed by the participant, leading to counting errors of grocery products during video coding. Nevertheless, we do not expect that this possibility may have affected all of our areas of interests similarly. For example, different products (AOI1) and products that were actually bought (AOI3) may suffer less from device slippage, whereas price tags (AOI2) may be affected stronger since price tags show a smaller surface compared to products. Further studies should consider actual accuracy, for example as described in Santini et al. [35].

Furthermore, we did encounter some challenges with respect to the data collection in the supermarket and the video coding. For example, the connection cable from the eye tracker glasses to the tablet had to be tapped down, otherwise the participants’ movements could have displayed the cable and thus interrupted the recording. It was sometimes difficult to recognize specific products during the video coding process because of the changing illumination in the supermarket. This feasibility study was almost unique among eye tracker studies in that we ran it in the wild and involved older adults rather than undergraduate students. Hence, some helpful practical recommendations might be derived from our feasibility study. These recommendations may be useful for future studies that plan to innovatively expand the traditional methods repertoire of personality science by using eye tracking in a real-life setting.

### Practical Recommendations

1. Using the wireless Dikablis Professional Eye Tracking Glasses, it was no problem to record eye movements if participants wore glasses (reading glasses or varifocals).

2. As participants move freely during grocery shopping, the connection cable from the eye tracker glasses to the tablet should be tapped down so that it cannot be displayed and thus interrupt the re-cording.

3. The WLAN connection might disrupt if the dis- tance from the participant to the router is too large (what easily can happen if the supermarket is big). In this case, the eye tracker data are stored on the tablet (offline) and can be downloaded later. However, it is not possible to monitor the cameras on the laptop in real-time if there no WLAN connection.

4. The video coding is very time-intensive if no markers are used. Depending on the area of interest that is coded, it may last up to 24 hours and more to code a video of 10 minutes. For a sample with enough power (i.e., >84 participants), data coding might require approximately 2,016 hours. Videos should be double-coded to provide an inter-rater reliability.

5. The illumination in the supermarket may change in different sections. Furthermore, some packing colors (e.g., red) may interact with the illumination. Both of these factors may influence the quality of the video material. In turn, this may cause difficulties to recognize specific products during the video coding process and lead to a weaker inter-rater reliability.

6. Participants should pay in cash rather than by credit card (if it is a chip card) as the scene camera will record where they look at, that is the numeric keypad (PIN code).

7. Based on our power analysis (see “Statistical Analyses”), we recommend that future studies sample at least 84 participants. This size is required to detect correlation coefficients around *r* = .30 (Cohen’s *d* = 0.63). However, to detect weaker associations (i.e., *r* = .05, Cohen’s *d* = 0.1), a sample of 3,136 participants is needed.

8. Lastly, the articles of Cognolato, Atzori, and Müller [36] as well as Santini and colleagues [35] may be helpful guidelines when planning an eye tracker study in a realistic setting.

### Future Directions

The focus of the present study was on the feasibility of the real-life assessment paradigm. Now, as this first and critical step has been taken, there is abundant room for further research. Future studies including larger samples and possible covariates are needed to determine whether there are no associations, or under which circumstances different personality traits and eye movements are related in real life. Moreover, one potential line of future research might be to develop an automated coding method - for example using artificial intelligence or object detection [37] - to accelerate the eye tracker data evaluation, especially if large samples will be investigated.

To conclude, using eye tracking in real life might yield some challenges such as a very time-intensive video coding process, however, it may also open up new avenues for personality and aging research. The present feasibility study emphasizes that grocery shopping as a real-life assessment paradigm is suitable for older adults. Furthermore, it provides important insights into the eye tracker data collection in real life and may have formed some basis for future research.

## Ethics and Conflict of Interest

The authors declare that the contents of the article are in agreement with the ethics described in http://biblio.unibe.ch/portale/elibrary/BOP/jemr/ethics.html and that there is no conflict of interest regarding the publication of this paper.

## Acknowledgements

This research was supported by a grant to the first au- thor from the Jacobs Foundation. During the work on her dissertation, Damaris Aschwanden was a fellow of the International Max Planck Research School on the Life Course (LIFE, www.imprs–life.mpg.de).

We thank the former student assistants Stefanie Jakob, Stefanie Lindner, Pia Neuschwander, and Merve Özonar for their great work in this feasibility study.
